# Increasing access, equitability, and rigor in the assessment of Neighborhood Mortgage Discrimination

**DOI:** 10.21203/rs.3.rs-4419606/v1

**Published:** 2024-05-23

**Authors:** Leah Moubadder, Maya Bliss, Maret Maliniak, Hannah Waddel, Jeffery Switchenko, Howard Chang, Michael Kramer, Lauren McCullough

**Affiliations:** Emory University Rollins School of Public Health; Emory University Rollins School of Public Health; Emory University Rollins School of Public Health; Emory University Rollins School of Public Health; Emory University Rollins School of Public Health; Emory University Rollins School of Public Health; Emory University Rollins School of Public Health; Emory University Rollins School of Public Health

## Abstract

Mortgage discrimination alters the distribution of investment, opportunity, and economic advantage—key contributors of health disparities. Leveraging Home Mortgage Disclosure Act data, we assessed mortgage denial risk in 380 U.S. urban areas. We estimated the risks by census tract–relative to the urban-specific average—using a Bayesian spatial model with conditionally autoregressive distributions fitted with integrated nested Laplace approximation. This approach borrows information through spatial and non-spatial smoothing, resulting in stable estimates in the presence of sparse data. The method, publicly accessible, allows researchers to apply our approach, fostering deeper insights into mortgage lending discrimination and systematic neighborhood disinvestment.

## Introduction

Access to homeownership and stable housing is pivotal for health and well-being.^1^ Yet, persistent bias in the housing market, despite federal anti-discrimination laws, differentially affects communities of color and low-income neighborhoods.^2,3^ This prejudice in mortgage lending not only hinders wealth accumulation and perpetuates housing racism, but diverts resources from underserved areas, leading to or sustaining neighborhood decline. Such discriminatory practices, reminiscent of historical redlining, create race and wealth segregation, impacting essential components of well-being like education and social capital. There is growing interest in assessing the health implications of mortgage discrimination but measuring mortgage discrimination can be challenging.

Study of patterns of mortgage discrimination could be population-centered (e.g. stratified by applicant race or ethnicity) or place-centered, and each provides important information. Recognizing the relationship between longstanding structural discrimination and contemporary racial residential segregation, we choose to focus on place-based estimation of mortgage denial given place-based stigma may be one mechanism guiding lenders decisions.

Bayesian methods for small area estimation are ideally suited to address several inter-linked analytic challenges including borrowing of spatially proximate statistical information to stabilize estimates in places with sparse data and incorporating spatial autocorrelation into estimates. We used R-INLA to approximate Bayesian posterior distributions of mortgage denial.

This study aims to quantify area-level mortgage denial risks using publicly accessible Home Mortgage Disclosure Act Data (HMDA) across metropolitan areas in the United States. To account for sparse and dependent data, we used a Bayesian hierarchical spatial model to measure mortgage denial risk within a census tract compared to the overall average of the respective metropolitan area. This approach can be easily applied and is conducive to large spatial datasets, thus removing a potential barrier for those interested in exploring access to homeownership. This metric is freely available on GitHub to encourage collaborative efforts across disciplines to address neighborhood discrimination in mortgage lending.

## Methods

We developed a metric to measure mortgage discrimination using 2010–2014 data from the HMDA. This approach was informed by previously published methodologies by Gee et al.^4^, Mendez et al.^5^, and Beyer et al.^6^. The HMDA was enacted by Congress in 1975, requiring that financial institutions collect and publicly disclose information on their mortgage lending practices, which includes information at the loan-level. Variables that are relevant to this analysis that are reported in the HMDA data are mortgage loan outcomes (e.g., approved), type, and purpose (e.g., home purchase), applicant sex, and the census tract of the property for which the loan was applied.

We estimated the Global Moran’s I for each MSA to assess spatial autocorrelation. For our main analysis, we estimated the relative risk of mortgage denial within each census tract compared to the average of the respective MSA using a hierarchical Bayesian spatial model. The hierarchical component of this model explicitly accounts for the clustering of loans within the same census tract and potential correlation between census tracts within the same MSA. Moreover, it allows adjustment by individual level (i.e., loan-level) covariates, such as loan-to-income ratio and applicant sex, while estimating residual census-tract level risk of denial. We employed a Besag, York, and Mollie (BYM) model to incorporate these considerations.^7^ The BYM model decomposes the census-tract spatial residual random effect into a sum of an unstructured random effect modeled using a normal distribution, and a spatially structured random effect modeled using a conditional autoregressive specification (CAR). The BYM model assumes that census tract-specific risks of mortgage denial may vary from one another and that those differences can be described by estimating random effects that explicitly account for spatial relatedness and random effects that are spatially independent.^8^

We assumed a binomial likelihood for the number of denials within each tract *Y*_*ij*_, with a probability of loan denial, *θ*_*ij*_, for individuals indexed by *i* within census tract *j*.

$$Y_{ij}∼\text{Binominal}(n_{ij},\theta_{ij})\;\text{logit}(\theta_{ij})=a+H_{ij}\beta_{j}+u_{j}+v_{j},$$

where *α* is the overall average baseline risk in the metropolitan area, *H*_*ij*_ denotes a vector of loan-level fixed covariates (loan-to-income ratio and applicant sex) and their associated coecients *β*_*j*_. Random effects *u*_*j*_ and *v*_*j*_ are spatially structured and unstructured residuals, respectively, for each census tract. We specified a CAR prior^7^ for *u*_*j:*_

$$u_{g}|u_{j,g\neq j}∼N\frac{\sum_{j}w_{gj}u_{j}}{\sum_{j}w_{gj}},\frac{\sigma_{u}^{2}}{\sum_{j}w_{gj}}$$

Where *w*_*gj*_ are the weights defining the relationship between area *j* and its neighbors and the conditional mean is a weighted average of the other *u*_*j*_. The adjacency matrix is specified such that *w*_*gj*_ = 1 if area *g* and *j* share a boundary (e.g., queen contiguity) and *w*_*gj*_ = 0 otherwise. The parameter *v*_*j*_ is modelled using an exchangeable Normal prior,

$$v_{j}∼N(0,\sigma_{v}^{2})$$

Finally, we specified a diffuse Normal prior *β*_*j*_ ~ *N* (0, 1000) for the fixed effects.

We approximated the posterior means and 95% credible intervals using Integrated Nested Laplace Approximation (INLA; R-package: http://www.r-inla.org/). The parameters of interest for this metric are the area-level residuals, which are represented by the sum of the random effects (i.e., *uj* + *vj*). When exponentiated, this value represents the relative probability (e.g. a relative ratio) of loan denial for a given census tract as compared to the MSA at large, controlling for loan-level covariates. A relative ratio greater than 1 indicates a census track has an increased risk of mortgage loan denials, while a value between 0 and 1 indicates a decreased risk.

To be included in this analysis, applications must have been for home purchase loans, one to four-family or multifamily properties, and owner-occupied principal dwellings. We categorized loans as approved if a loan received one of the following determinations: loan originated, application approved but not accepted, preapproval request approved but not accepted, and loan purchased by the institution. Loans or preapprovals that were denied by the financial institution were categorized as loan denials, and withdrawn applications or files closed for incompleteness were excluded. Additionally, mortgage applications with missing values for relevant variables, such as state code, were excluded. All results and code are publicly available at https://github.com/LM0331/Mortgage-Discrimination-Measure.

## Results

After instituting our eligibility criteria, there were 16,816,010 mortgage applications across 60,399 census tracts nested within 380 MSAs in the contiguous US used to calculate our measure. Mortgage denial was relatively rare (10.3%). The median number of applications per MSA was 13,736 (IQR 7,500 – 35,197).

**Figure 1:**
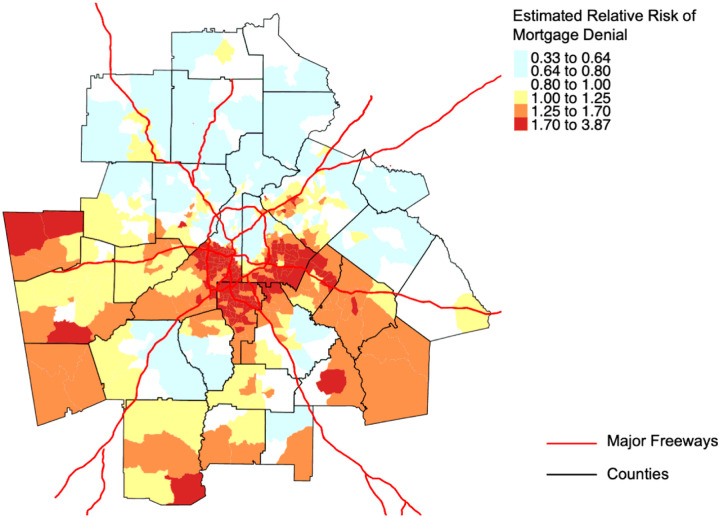
Census tract variation in the relative risks of mortgage denial in Metropolitan Atlanta, Georgia, adjusting for applicant sex and loan-to-income ratio. The map shows areas of high risk (red) and low risk (blue) of experiencing differential denials in mortgage lending.

Statistically significant spatial dependence of mortgage denial risk was detected in 356 of 380 (94%) of MSAs and the majority of the Moran statistics indicated positive spatial autocorrelation (median 0.24, IQR 0.11–0.35). We observed geographic variation in mortgage discrimination across MSAs and estimates tended to be precise (median 0.18, IQR 0.15–0.22). Figure 1 highlights Atlanta as an example, where census tracts clustered in the southern region experience a higher risk of mortgage denials. The MSA that exhibited the highest proportion of census tracts with an elevated risk of mortgage denial was Gainesville, GA (64%), while Chambersburg-Waynesboro, PA, San Angelo, TX, and Winchester, VA-WV had the lowest proportions (33%).

## Discussion

In this study, we aimed to investigate the relative risk of neighborhood level mortgage denial across 380 metropolitan areas in the contiguous US. To achieve this, we employed a Bayesian spatial model to account for the inherent spatial dependence in mortgage denial data. Our findings revealed intra-urban spatial heterogeneity and in the risk of mortgage denial, shedding light on the complex dynamics that influence lending practices and housing access within different MSAs. While some census tracts exhibited higher risks of mortgage denial, others did not, indicating the presence of localized factors that influence lending decisions. The identification of such spatial patterns is essential in understanding the underlying drivers of disparities in mortgage approval rates and potential downstream effects related to neighborhoods and public health. Uncovering distinct patterns of mortgage denial risk can provide valuable insights for policymakers, urban planners, and public health professionals. These data, code, and methodology have been made publicly available on Github for researchers to utilize and shape to their needs.

Discrimination in mortgage lending at the area-level systematically inhibits a source of neighborhood investment, which can be detrimental to communities that are already low-resourced or experiencing neighborhood decline. We postulate that this persistent and intentional disinvestment is one of many forms of institutional discrimination upstream of spatial inequalities in harmful exposures (e.g., air pollution^9^). Moreover, although it hasn’t been explored in this specific analysis, there is a high likelihood that neighborhoods facing a greater risk of mortgage denial are subject to discrimination based on their racial composition or socioeconomic status. This is substantiated by prior investigations into factors influencing loan approvals, which have considered applicant race and lenders’ perceptions of socioeconomic status as predictors, irrespective of creditworthiness.^2,10,11^ Homeownership can be used as one tool to improve neighborhood stability, safety, and involvement in local democratic institutions in what are otherwise low-opportunity areas.^3^ This underscores the importance of place-based initiatives to expose or reduce potential discriminatory elements in local real estate markets and lending practices.

There are two important limitations to this metric. First, the HMDA data has missing values. We conducted a complete case analysis, thus assuming that missingness is occurring completely at random. However, if this is not the case, these results may be biased.^12^ Relevant values for this analysis, such as income, are reported by lending institution’s staff or via an automated collection process.^13^ Assuming that institutions would have complete data on income amount to make an application determination and that missingness in the HMDA data occur via errors in the automated algorithm or human error, we find it unlikely that data are missing not at random, which is often considered the most problematic.^14^ A future investigation should explore patterns of missingness within these data, which would be a particularly important endeavor considering the increasing interest in using HMDA data in health research. Second, given the availability of the HMDA data, we conducted these analyses at the census tract level. While providing this measure at the census tract level is useful for linking to external datasets, it is likely that lending institutions discriminate according to colloquial neighborhood boundaries, which would result in spatial misalignment with census geographies, rather than census tract boundaries.

This study focused on estimating area-level access to mortgage loans across all 380 metropolitan areas. Utilizing a Bayesian spatial model, we addressed both spatial and statistical dependencies inherent in these data, while accounting for differences in the prospective borrowers’ risk by adjusting for loan-level covariates. This modeling approach was implemented using a computationally-efficient method, ensuring the scalability and accessibility of the metric. By providing a robust and transparent method for mortgage discrimination, our novel approach can inform interventions to promote equitable lending practices in these areas. The persistence of differential mortgage approvals by place, even five decades after the enactment of the 1968 Fair Housing Act, underscores the need for more extensive interdisciplinary inquiries. Such investigations are crucial to understanding the potential health inequities that may have arisen due to the lack of transparency and accountability in the enforcement of fair housing policies. By making the analytic approach publicly available, researchers will be able to adapt our methods to their needs and advance the understanding of the impact of mortgage discrimination on affected communities. This will facilitate the replication and extension of our findings and encourage collaborative research efforts to address this pressing issue.
